# Automated recognition and segmentation of lung cancer cytological images based on deep learning

**DOI:** 10.1371/journal.pone.0317996

**Published:** 2025-01-31

**Authors:** Qingyang Wang, Yazhi Luo, Ying Zhao, Shuhao Wang, Yiru Niu, Jinxi Di, Jia Guo, Guorong Lan, Lei Yang, Yu Shan Mao, Yuan Tu, Dingrong Zhong, Pei Zhang

**Affiliations:** 1 Department of Pathology, Chengdu Second People’s Hospital, Sichuan, China; 2 Department of Pathology, China-Japan Friendship Hospital, Beijing, China; 3 Technical University of Munich, Munich, Germany; 4 School of Optics and Photonics, Beijing Institute of Technology, Beijing, China; 5 Key Laboratory of Photoelectronic Imaging Technology and System, Ministry of Education, Beijing Institute of Technology, Beijing, China; 6 Thorough Lab, Thorough Future, Beijing, China; 7 Chengdu Uniwell Medical Laboratory, Sichuan, China; Affiliated Hospital of Nanjing University of Chinese Medicine: Jiangsu Province Academy of Traditional Chinese Medicine, CHINA

## Abstract

Compared with histological examination of lung cancer, cytology is less invasive and provides better preservation of complete morphology and detail. However, traditional cytological diagnosis requires an experienced pathologist to evaluate all sections individually under a microscope, which is a time-consuming process with low interobserver consistency. With the development of deep neural networks, the You Only Look Once (YOLO) object-detection model has been recognized for its impressive speed and accuracy. Thus, in this study, we developed a model for intraoperative cytological segmentation of pulmonary lesions based on the YOLOv8 algorithm, which labels each instance by segmenting the image at the pixel level. The model achieved a mean pixel accuracy and mean intersection over union of 0.80 and 0.70, respectively, on the test set. At the image level, the accuracy and area under the receiver operating characteristic curve values for malignant and benign (or normal) lesions were 91.0% and 0.90, respectively. In addition, the model was deemed suitable for diagnosing pleural fluid cytology and bronchoalveolar lavage fluid cytology images. The model predictions were strongly correlated with pathologist diagnoses and the gold standard, indicating the model’s ability to make clinical-level decisions during initial diagnosis. Thus, the proposed method is useful for rapidly localizing lung cancer cells based on microscopic images and outputting image interpretation results.

## Introduction

Lung cancer is the leading cause of cancer-related deaths globally [1]. Recent advances in computed tomography (CT) screening technology and the expansion of health screening have enhanced the early detection of minor lung lesions [2, 3]. However, as lung cancer is usually asymptomatic, many patients are diagnosed at an advanced stage; thus, the optimal period for surgical treatment is often missed, and lymph node and distant metastases are developed [4, 5]. Cytology is a simple and rapid method that is less invasive and provides better preservation of complete morphology and detail than histological examination of surgical and biopsy samples. Lung cancer cytology specimens are collected in various manners, including sputum, transbronchial needle aspiration biopsy, bronchoscopy brush examination, bronchoalveolar lavage fluid, imprint smear, and pleural effusion [6–9]. However, traditional cytological diagnosis requires an experienced pathologist to evaluate all sections individually under a microscope, which is a time-consuming process with low interobserver consistency [10]. Therefore, improving the diagnostic accuracy and consistency remains challenging, and inexperienced pathologists are more likely to provide misdiagnoses and missed diagnoses [11].

Artificial Intelligence (AI) technology has evolved from expert systems to traditional machine learning, and subsequently, deep learning (DL). Most studies on AI cytology have used traditional machine learning, which requires extensive knowledge of feature mapping and descriptions to handcraft features and detect objects from images [12]. In addition, the unpredictability of the model evaluation results and the uncontrollability of the decision-making process hinder the further application of such methods in practice. DL significantly outperforms traditional machine learning in natural image processing and has achieved better performance in visual computing and editing applications [13].

Over the past several years, AI has made significant advances in the automated segmentation of medical images, including the localization of tumor lesions in MRI images, assessment of cardiac function, and identification of metastatic hotspots in bone single-photon emission CT images [14–16]. The required time and bias of manual tumor screening can be reduced by annotating regions of interest in an automated and repeatable manner [17]. However, most automatic segmentation models cannot meet clinical requirements owing to performance limitations and the pixel image processing speed [18]. Therefore, the rapid identification and accurate classification of lesions in medical images are problems that need to be solved [19].

You Only Look Once (YOLO) is a new DL object-detection algorithm that can mark every instance in an image by segmenting each instance at the pixel level, and it completes localization and prediction simultaneously [20]. YOLOv8, which is the most recent YOLO series and the fastest algorithm for image recognition in AI, has been applied extensively in various areas including agriculture [21] and obstacle detection [22]. In the medical field, Rašić et al. [23] demonstrated that a model based on YOLOv8 has a remarkable ability to automatically detect and segment radiolucent lesions in the mandible.

We developed a lung cancer cytological recognition and segmentation model based on the YOLOv8 algorithm to improve the efficiency and accuracy of localizing malignant cells in lung cancer cytology images and compared the model results and pathologist interpretations. The highlight of this model compared to previous ones [11, 24] is the presentation of automatically localized malignant cells in the output image, which can be used for clinical-level decision-making during initial diagnosis.

## Materials and methods

### Data collection

A total of 405 lung lesion scratch-imprint cytology (SIC) samples and 205 normal lung tissue SIC samples were collected at China-Japan Friendship Hospital from February 2021 to April 2022 to develop and evaluate our lung cancer cytological recognition and segmentation model. The collection of tissue was approved according to ethical guidelines. All patients have obtained written informed consent, with all data anonymized.

Specifically, the lung lesion tissue and normal samples were collected simultaneously during the acceptance of the intraoperative pathology consultation. The tissue samples were fixed in 10% formalin and then dehydrated and embedded in paraffin for final diagnosis. The process of preparing the lesion SIC samples was as follows: Intraoperatively obtained tissue samples were immediately sent to the pathology department. First, the tissue was cut to expose the maximum extent of the lesion. The sample was scraped with the tip of a glass slide, following which the scraped surface was immediately evenly spread on another clean slide. The normal lung tissue SIC was created using the same method in the non-tumor area of the specimen. The slides were immersed in 95% ethanol for fixation and then stained with hematoxylin and eosin (Yili Pathology Staining Company, Beijing, China). All slides were examined independently by two pathologists to determine whether they represented benign or malignant lesions. When the pathologists had different opinions, the final diagnosis was made by another adjudicating pathologist and verified by the pathological results of the postoperative formalin paraffin-embedding (FFPE) tissue.

### Data distribution and image annotation

A total of 4610 images, consisting of 2279 malignant images and 2331 non-malignant images in JPEG format, were collected using a microscope (Eclipse Ni, Nikon, Japan) attached to a digital camera (D-CleverEye, Dipath, Hangzhou, China) at 20× magnification. The images were divided into training, validation, and test sets at a ratio of 8:1:1. We used the training set data to train the model, the validation set to verify the generalization ability of the model, and the internal test set to evaluate the effectiveness of the model in the instance segmentation task. Furthermore, 311 SIC images, 120 pleural fluid cytology images, and 120 bronchoalveolar lavage fluid cytology images were used as additional test sets (Table 1) to evaluate the effectiveness of the model in the image-level binary classification task (malignant or benign). Additionally, cytological images of malignant lesions from the additional test sets were categorized into four subtype groups: adenocarcinoma (n = 289), squamous carcinoma (n = 33), small cell carcinoma (n = 11), and others (n = 13). The initial matrix size of each JPEG image was 1920 × 1080 pixels.

**Table 1:** Number of images in each dataset.

	Training set	Validation set	Test set	Additional test set 1	Additional test set 2	Additional test set 3
Malignant	1814	226	239	226	60	60
Non-malignant	1875	235	221	85	60	60
Total	3689	461	460	311	120	120

Notes: Additional test sets 1, 2, and 3 denote SIC, pleural fluid cytology, and bronchoalveolar lavage fluid cytology test sets, respectively.

### Image preprocessing

To enhance the generalization and robustness of the model, this study employed various data augmentation techniques, which not only increase the diversity of the training data but also effectively mitigate the risk of overfitting. The specific data augmentation techniques include the following:

- **Color Jittering**: This technique involves randomly adjusting the brightness, contrast, saturation, and hue of the images to simulate different lighting and environmental conditions, thereby improving the model’s adaptability to color changes.
- **Noise Addition**: Adding Gaussian or other types of noise to the images enhances the model’s robustness against noise.
- **GridMask**: This technique randomly occludes parts of the images, simulating occlusion scenarios and improving the model’s ability to detect partially obscured objects.

The malignant areas of 2606 images in the training set were annotated by the pathologists mentioned in Section 2.1. Following annotation, a JSON file that contained the labels and coordinates of the contours corresponding to each slice was generated. Subsequently, the JSON file was transferred into a TXT file as the labels of each image.

### Model training and visualization of results

YOLOv8 is a DL-based instance segmentation algorithm that combines the concepts of object detection and semantic segmentation to label each instance in an image by segmenting the image at the pixel level.

We constructed our instance segmentation model based on YOLOv8x with the Darknet [25] architecture as its backbone. The model was implemented in PyTorch (1.13.0+cu117) using the Adam optimizer [26]. The weights of the model were evaluated on the validation set, with only the best and last ones saved.

We introduced heatmaps to visualize the model prediction results. The heatmap method is based on the attention mechanism, indicating the classification confidence of each pixel. The confidence was estimated based on the output features of the convolutional layer: a brighter color on the pixel results in higher confidence being assigned to that pixel.

**Fig 1:**
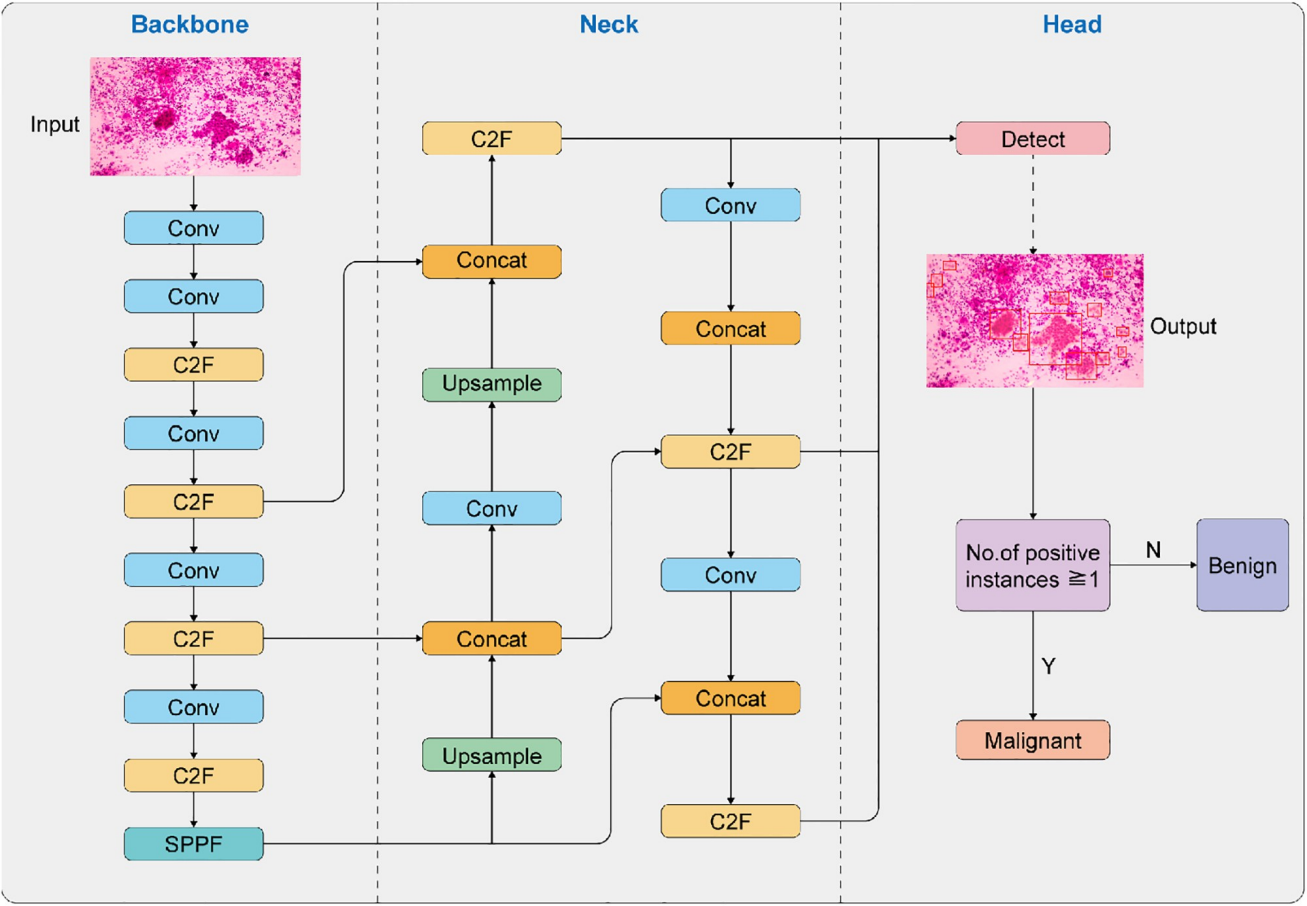
Model training and testing for diagnosis of pulmonary intraoperative cytology.

The image-level diagnostic criteria were as follows: (1) If the image is categorized as malignant, it contains at least one positive instance. (2) If the image is categorized as benign, it does not contain any positive instances. Fig 1 depicts the YOLOv8 network structure and image-level interpretation method.

The SIC images were input, and the YOLOv8 deep neural network was used for detection and segmentation. The output image was categorized as malignant if it contained at least one positive instance.

### Statistical analysis and comparison with pathologist diagnoses

We evaluated the final performance of the model using the test set of SIC images. The pixel-level results were evaluated on the original test set, and the image-level predictions were evaluated on the additional test set of SIC images. The mean pixel accuracy (MPA) and mean intersection over union (mIoU) were employed to evaluate the recognition and segmentation effectiveness of the model at the pixel level. The MPA is the proportion of pixels in each category that are correctly categorized; the mIoU refers to the proportion of intersections and concatenations between the two sets of images (labeled and predicted) [27]. The model performance was compared with the diagnoses by pathologists from a large healthcare organization (P1) and primary hospitals (P2 and P3), as well as that by a pathology student. The pathologists and student drew conclusions (benign or malignant) and annotated the regions of interest at the image level. We verified the agreement of the model predictions with the diagnoses by the pathologists and student by calculating Kendall’s correlation τ value [28], which is a metric for determining the degree of correlation between variables, to assess the predictive performance of our model at the image level.

We used accuracy, sensitivity, specificity, and area under the receiver operating characteristic (ROC) curve (AUC) to describe the model effects. Accuracy reflects the closeness between the predicted and actual results. Sensitivity indicates the rate of positive samples in the model prediction results, whereas specificity indicates the rate of negative samples in the results. AUC represents the effect of the model running in the real world. In addition, we evaluated the generalization ability of the model on pleural fluid cytology and bronchoalveolar lavage fluid cytology images. We performed two-sample t-tests for all of the above metrics to determine whether statistically significant differences appeared between the results.

### Model improvement and innovation

In this study, we introduced a Squeeze-and-Excitation Attention (SEAttention) [29] layer into the backbone of YOLOv8-seg to enhance its performance in cell region segmentation of biomedical images. YOLOv8-seg, known for its real-time object detection and segmentation capabilities, is particularly well-suited for applications that require rapid and accurate predictions. However, more complex and detailed tasks, such as biomedical image analysis, necessitate a refined attention mechanism to capture subtle feature differences. This is where the SEAttention layer plays a crucial role by selectively enhancing the most important feature channels in the network.

The SEAttention layer operates by introducing channel-wise attention to help the model focus on the most relevant aspects of the input image. It begins by applying global average pooling to condense spatial information into a single value per channel. Subsequently, a fully connected network models the dependencies between channels and generates attention weights. These weights are then applied to recalibrate the feature maps, emphasizing channels that significantly contribute to accurate cell segmentation. The design of the SEAttention layer is efficient, adding only a small computational overhead, which makes it suitable for real-time applications of object detection and segmentation with YOLOv8.

The SEAttention layer’s ability to recalibrate feature maps by emphasizing informative channels while suppressing less useful ones is particularly beneficial in biomedical imaging, where cell structures may be faint or obscured by noise. This capability allows the model to focus on fine-grained details. By guiding the network to prioritize the most relevant features, the attention mechanism enhances the model’s accuracy in detecting and segmenting cells. Our results suggest that attention mechanisms, such as SEAttention, are valuable tools for improving the performance of DL models in complex segmentation tasks, particularly in biomedical image analysis, where precision and sensitivity are critical. Future work could explore the extension of this approach to other applications requiring fine-grained feature detection or segmentation, potentially leading to broader adoption in fields such as medical diagnostics, pathology, and bioinformatics.

## Results

The aim of this study was to identify malignant and benign samples and locate malignant regions in the images. We used 4610 images, which were derived from 610 SIC samples (405 lung lesion and 205 normal lung tissue samples). The diagnosis of postoperative FFPE samples was used as the gold standard. In the final diagnoses of the 405 lung lesion samples, 362 (89.4%) were determined to be malignant and 43 (10.6%) were determined to be benign. The baseline characteristics of the lung lesion samples are presented in S1 Table.

**Fig 2:**
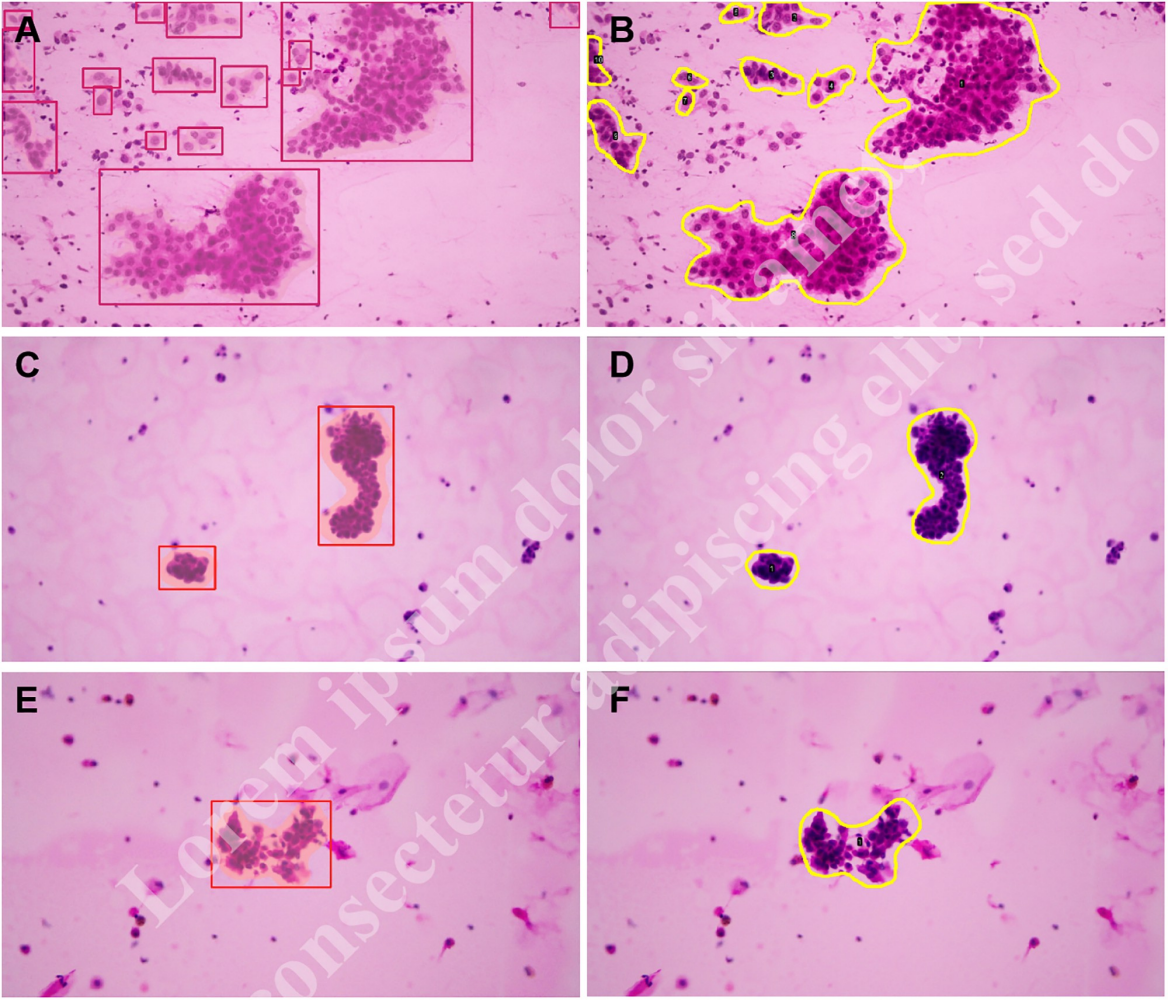
Example visualization of malignant regions detected by the model (A, C, and E) compared with malignant regions annotated by pathologists (B, D, and F). (A) and (B) are SIC images, (C) and (D) are pleural fluid cytology images, and (E) and (F) are bronchoalveolar lavage fluid cytology images.

At the pixel level, the MPA and mIoU of the model prediction on the test set reached 0.80 and 0.70, respectively. The images output by the model visualized its predictions at the pixel level, as illustrated in Fig 2. The red box shows the outer rectangle of the model identification contour, and the red-covered inner region represents the malignant region predicted by the model.

**Fig 3:**
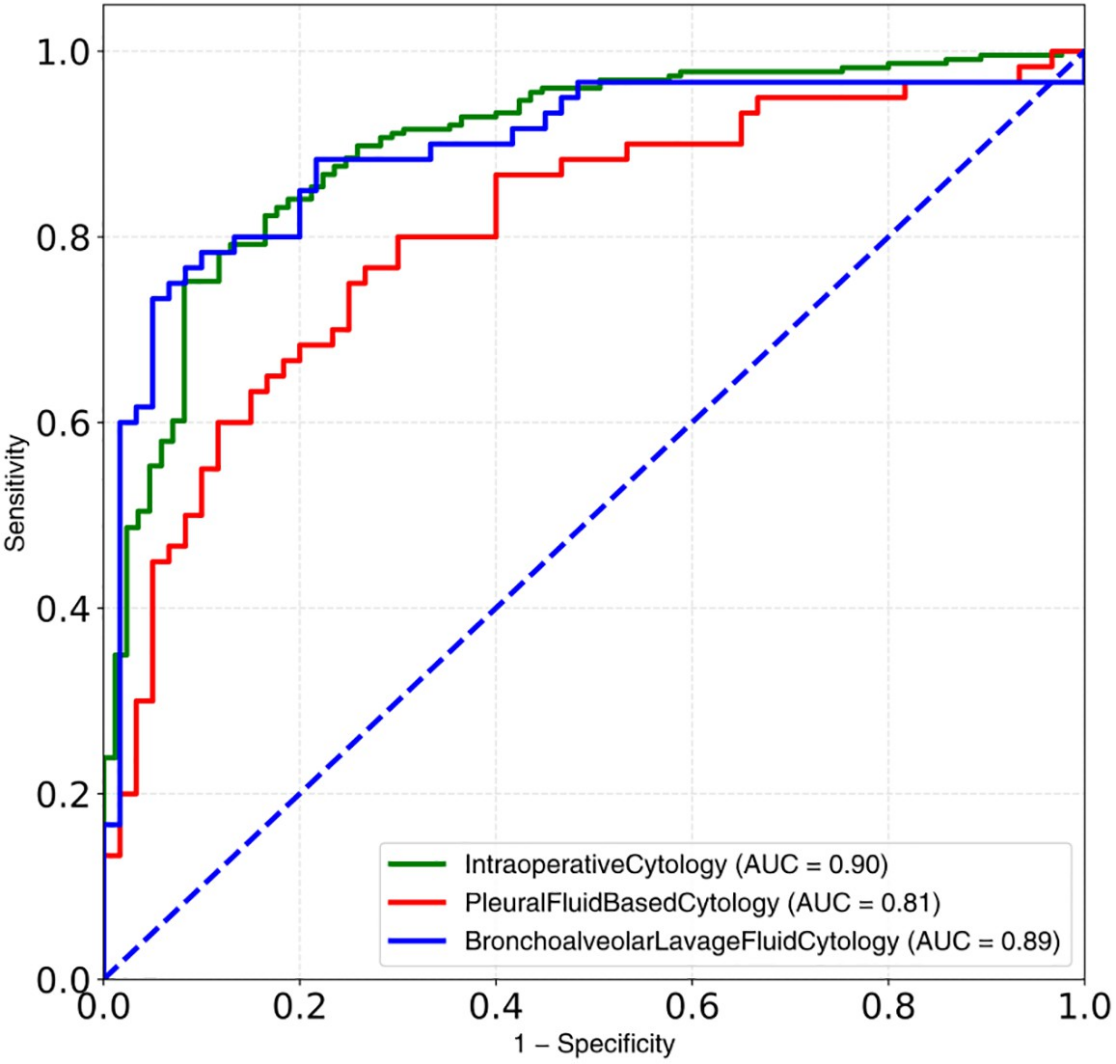
ROC curves of model on SIC, pleural fluid cytology, and bronchoalveolar lavage fluid cytology test sets.

We evaluated the predictive effectiveness of the model at the image level on the additional SIC test set. The accuracy, sensitivity, and specificity of the model for the image-level diagnosis prediction were 91.0%, 95.6%, and 78.2%, respectively, whereas the AUC was 0.90. We also tested the model on the additional pleural fluid cytology and bronchoalveolar lavage fluid cytology test sets, which yielded AUCs of 0.81 and 0.89, respectively (Fig 3). The model’s accuracy in identifying malignant cells among the subtype groups of adenocarcinoma, squamous cell carcinoma, and small cell carcinoma in these additional test sets was 92.7%, 97.0%, and 81.8%, respectively.

The comparison revealed that the malignant regions predicted by the model were approximately consistent with the regions of interest annotated by the pathologists (Fig 2). The accuracies of P1, P2, P3, and the student were 94.9%, 92.0%, 87.5%, and 72.7%, respectively.

**Fig 4:**
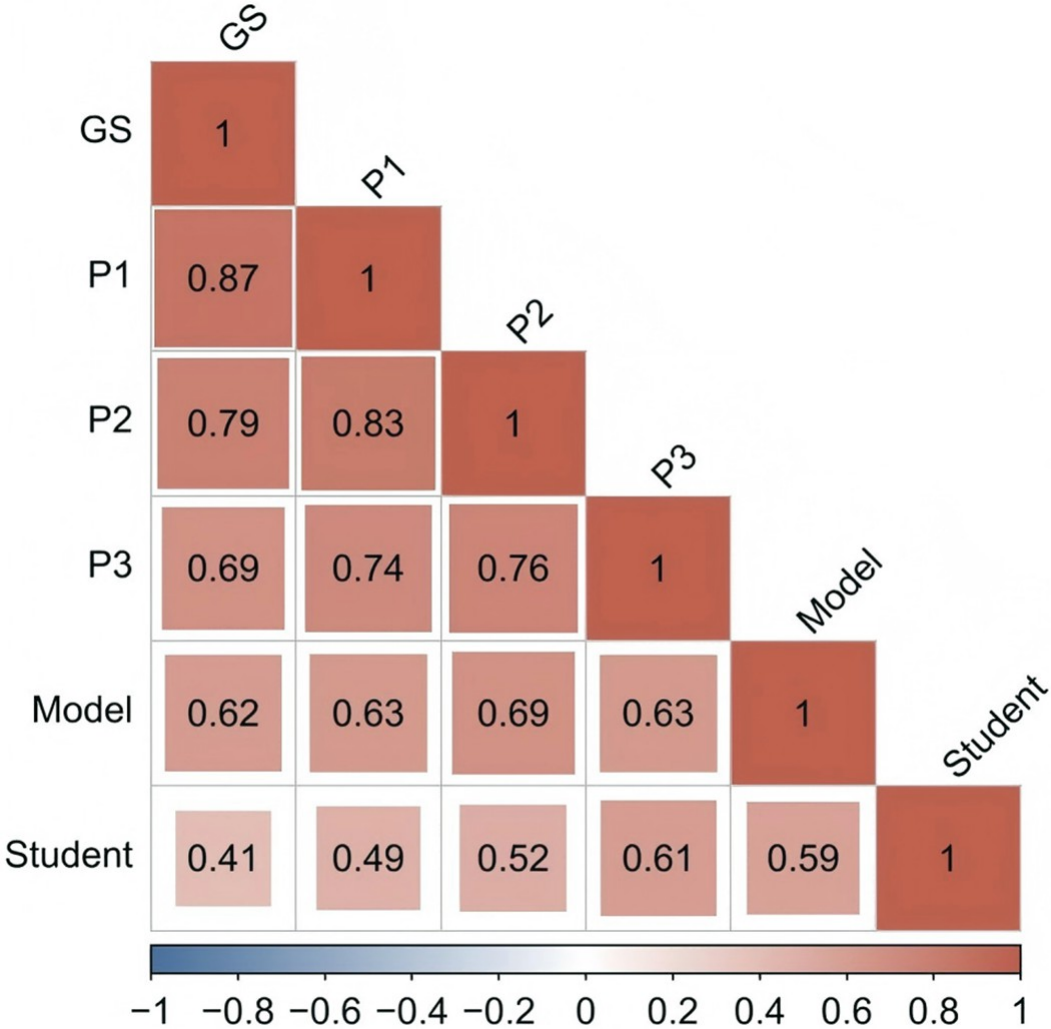
Kendall’s correlation τ values. GS: gold standard. P1, P2, and P3: pathologists from different institutions.

**Fig 5:**
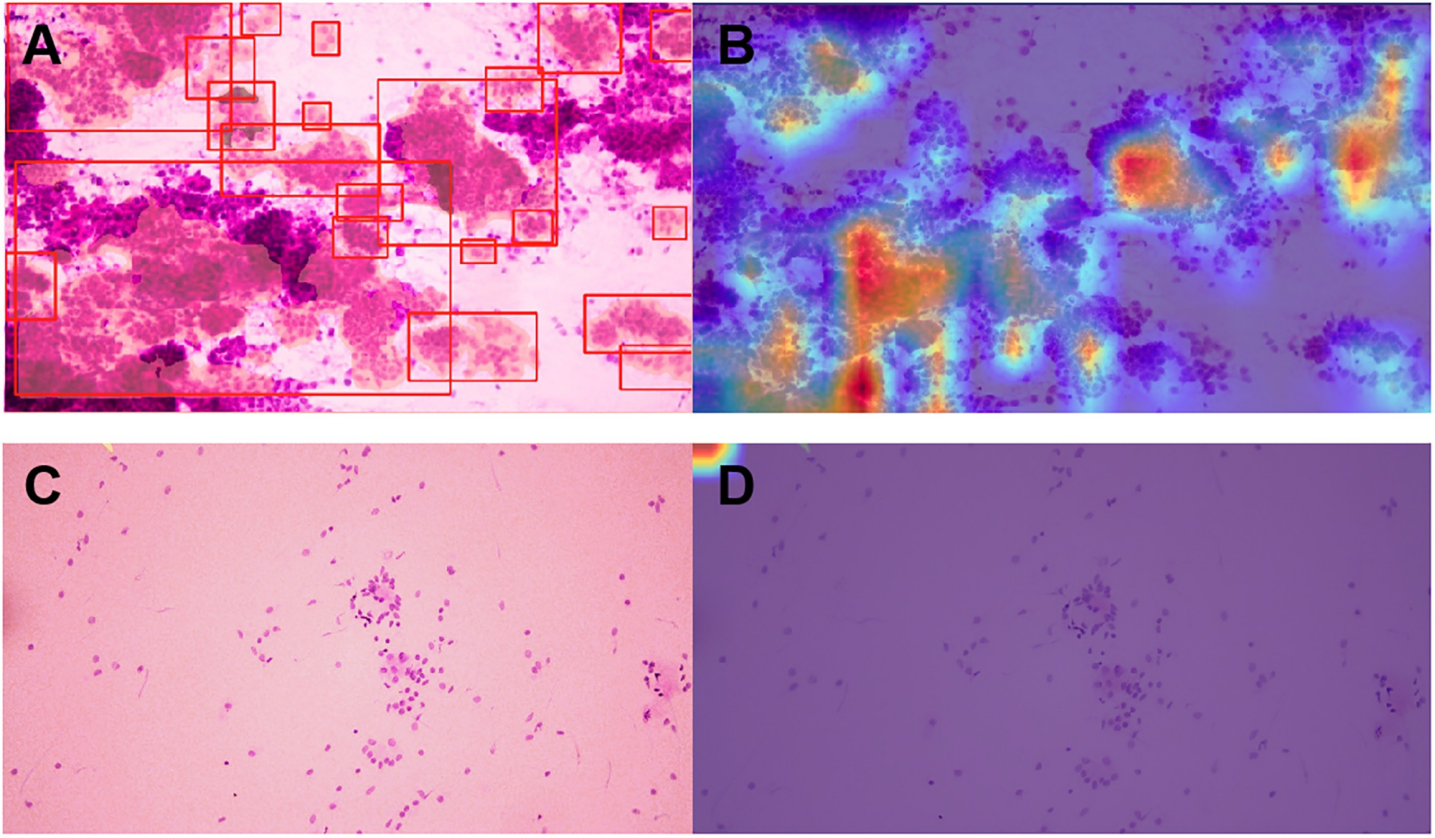
Visualization of model prediction results (heatmaps) indicating the classification confidence of each pixel. (A) Malignant images and (B) corresponding heatmap. (C) Benign image and (D) corresponding heatmap.

The correlations of the gold standard with the results of the pathologists, student, and model were analyzed separately. The results showed that the diagnoses by the pathologist in the large healthcare organization (P1) had the most significant correlation with the gold standard, with a τ value of 0.87 (*p* < 0.01). The diagnoses by the pathologists in the primary hospitals (P2 and P3) had a high correlation with the gold standard, with τ values of 0.79 (*p* < 0.01) and 0.69 (*p* < 0.01) for P2 and P3, respectively, whereas the diagnoses by the student had the lowest correlation with the gold standard, with a τ value of 0.41 (*p* < 0.01). The model predictions had a high correlation with the gold standard, with a τ value of 0.77 (*p* < 0.01) [28]. It can be concluded that the performance of our model was comparable to that of the pathologists and better than that of the pathology student (Fig 4). In addition, we introduced heatmaps to explain the results predicted by the model, as shown in Fig 5.

### Model comparison

MaskRCNN is a widely used and advanced model suitable for medical image segmentation tasks. In this study, we compared the performance of YOLOv8-SE (YOLOv8-seg with SEAttention) and MaskRCNN utilizing key metrics such as accuracy, precision, recall, and AUC (Table 2), each of which highlights various aspects of the models’ classification abilities.

**Table 2:** Comparison of key metrics between Yolov8 and MaskRCNN.

	Yolov8-SE	MaskRCNN
Accuracy	0.91	0.71
Precision	0.88	0.64
Recall	0.96	0.99
AUC	0.95	0.90

**Fig 6:**
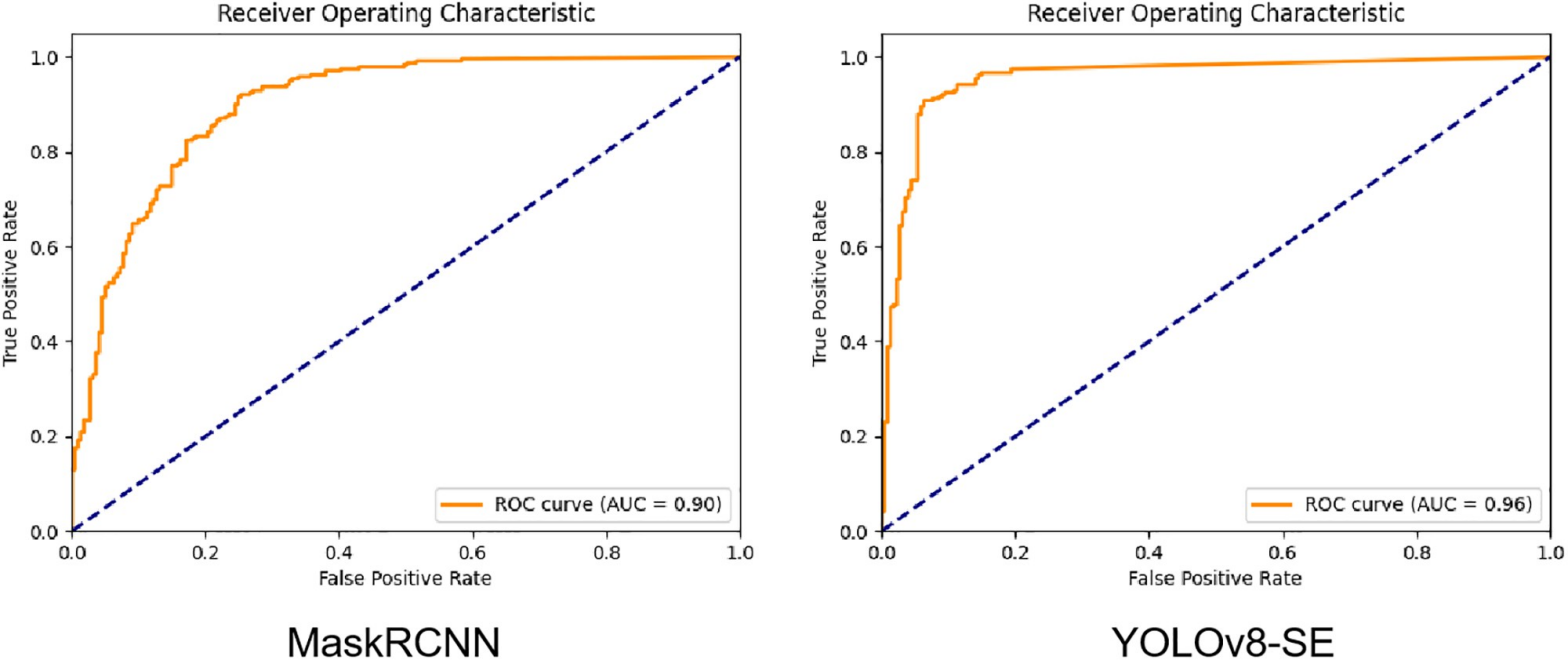
ROC curves for MaskRCNN and Yolov8-SE.

YOLOv8-SE demonstrated an adequate balance between precision and recall, achieving higher accuracy and AUC (Fig 6). This balance makes it well-suited for applications that require both high precision and recall without significantly compromising either, such as in object detection tasks where accurately detecting and identifying instances are critical. By contrast, MaskRCNN exhibited stronger recall but lower precision, indicating that it exceled at identifying all possible positives, even at the expense of producing more false positives. This trade-off may be more applicable in fields where minimizing false negatives is crucial (e.g., detecting all potential defects or medical conditions); however, it results in a higher number of false positives. Given its superior accuracy, precision, and AUC, YOLOv8-SE appears to offer a more versatile and reliable solution across a broader range of tasks. By contrast, MaskRCNN may be more specialized for scenarios that prioritize high recall, where missing a true positive is significantly more costly than incorrectly identifying a negative.

## Discussion

Cytological examination is the most appropriate technique for screening diagnosis of cancer. However, it is less accurate, more labor-intensive, and more time-consuming than histopathological examination. New techniques, such as AI image analysis, can enhance test accuracy while reducing labor and time costs [30].

AI plays a crucial role in solving various challenging tasks in medical pathology, including medical cytology. Most cytology AI studies to date have used traditional machine learning, which relies on the manual extraction of features and the selection of micrographs or image blocks of representative cells [31]. Some recent studies have applied DL to cytology diagnostics, achieving high levels of model accuracy and sensitivity [32–34] (S2 Table). Teramoto et al. [11] developed an automated classification scheme for lung cancers from cytology images based on 76 samples and achieved an accuracy of 71.1%. They then developed a VGG-16 CNN model that attained classification accuracies of 79.2% on a 40-case dataset [35] and 85.3% on a 60-case dataset [36]. Gonzalez et al. [37] used 40 cases to train an Inception V3 model to distinguish between small-cell lung cancer (SCLC) and large-cell neuroendocrine carcinoma (LCNEC), achieving AUC values of 100%, 100%, and 87.5% on the Diff-Quik staining dataset, PAP staining set, and H&E staining set, respectively. Xie et al. [24] applied a DL model for classifying benign and malignant cases based on lung cytological images using 404 samples, and the results were comparable to the average diagnostic level of cytologists. Kim et al. [38] developed a DL model for respiratory cytology using 1,273 whole slide images (WSIs) from a quality-control national dataset, achieving accuracy, sensitivity, and specificity values of 96.9%, 95.9%, and 98.2%, respectively. Our work included a large dataset and a wide coverage of 405 cases compared to previous studies, including 16 types of malignant and benign lung diseases.

We developed a lung cancer cytological segmentation model based on the YOLOv8 algorithm. YOLO recognizes objects in complex images by framing detection as a regression problem with spatially separated boundaries and class probabilities, and it is well known for its impressive speed and accuracy [39, 40]. In the medical field, Zhang et al. [41] established an automatic detection model for microaneurysms in fluorescein angiography images based on the YOLOv8 algorithm, with an accuracy and average precision of 0.98 and 0.95, respectively. Sahoo et al. [42] applied the YOLOv8 model for the automatic localization of colorectal cancer in CT images, achieving mean average precision, sensitivity, and accuracy values of 0.98, 0.83, and 0.96, respectively. A limitation of traditional cytopathology diagnosis is that a pathologist is required to scrutinize each section under the microscope, which is a time-consuming process with low interobserver consistency [10]. Therefore, owing to its powerful computational capabilities and fast and accurate target recognition, the YOLOv8 algorithm is well-suited to the rapid detection and localization of malignant cells in large, high-resolution cytopathology images and can increase the interpretability of the diagnostic result (malignant or benign) at the image level. To the best of our knowledge, our study is the first to apply the YOLOv8 algorithm to lung cancer cytopathology recognition, segmentation, and diagnosis. This model realizes real-time recognition and segmentation of images, as demonstrated by its ability to locate suspicious areas rapidly and reduce the time for detecting target cells.

We evaluated the recognition and segmentation effectiveness of the model on a test set. At the pixel level, the model achieved MPA and mIoU values of 0.80 and 0.70, respectively, and automatically annotated malignant cell regions in the output image. Subsequently, we verified the image-level diagnostic performance of the model on an additional test set of 311 SIC images and compared the interpretation differences with those of pathologists. The AUC of the model was 0.90 at the image level. We also tested the model on additional test sets of pleural fluid cytology and bronchoalveolar lavage fluid cytology images, resulting in AUCs of 0.81 and 0.89, respectively. Moreover, the model demonstrated good recognition of malignant cells across various histological types of cytological images. The results obtained on the additional test sets demonstrate that the model has a strong generalization ability and robustness.

The model predictions exhibited high correlations [43] with pathologist diagnoses and the gold standard (ranging from 0.66 to 0.80), which suggests that the model interpretations were logical. Compared to the gold standard, the accuracy of the model predictions (91.0%) ranged between the accuracy of the diagnoses made by all pathologists (from 87.5% to 94.9%) and the Kendall’s correlation τ value (τ = 0.77), which suggests that the model allows clinical-level decision-making during initial diagnosis.

YOLOv8-SE provides a more balanced approach than MaskRCNN, making it better suited for general classification tasks. By contrast, MaskRCNN may be preferred for tasks that demand extremely high sensitivity, even despite lower precision.

A total of 44 images were misclassified by the model in the additional test set of SIC images (S1 Fig). The model effectively classified images with direct cell morphology and distinctive features. Poorly adherent malignant cells with a scattered distribution were not easily recognized by the model [44], and malignant cells covered by inflammatory cells could also lead to misclassification. In addition, hyperplastic bronchial epithelial cells and clustered inflammatory cells could be misidentified as malignant. Although the model indicates the possible presence of malignant cells in the output images, the final diagnosis needs to be issued by a pathologist. We visualized the model predictions using heatmaps, which further improved the credibility of model interpretation and may enhance the diagnostic confidence of pathologists.

Our study has several limitations. WSIs, which encompass all the biological information present in cells, may be a more suitable domain for implementing DL models. However, the various techniques for preparing cytological slides, such as liquid-based thin-layer cytologic preparation or smear preparation, along with the three-dimensional nature of cytological images contribute to challenges related to overlapping and uneven cell distribution. These factors hinder the acquisition of complete and clear slide images [45–47]. Z-stacked scanning could enhance the accurate interpretation of cytology cases by scanning the same slide multiple times at different focal planes and stacking the images into a final composite. However, this technique requires additional time to train pathologists, and the large datasets necessitate substantial server support [48]. The acquisition of cytological images is also affected by sampling variability and inadequacy. The SIC images used in this study, obtained directly from the surface of the lesion, were chosen as the primary dataset because they exhibit typical cytologic morphology with abundant cellularity. The trained model yielded good results on the test sets. However, because the SIC specimens are not part of routine cytological examinations, and despite complementing the performance of the model with pleural fluid and BAL images, further validation of the model’s potential use with other common sample types (e.g., sputum, fine-needle aspiration cytology, etc.) is necessary. Currently, no grand challenges or extensive collections of lung cancer cytology images are publicly available in China. The dataset used in this study includes a diverse range of sample types and lesion subtypes, initially confirming the model’s generalizability. However, all datasets are still sourced from a single institution. Future studies will require data from different institutions, ethnicities, and countries to further validate the accuracy and scalability of the model.

## Conclusion

We developed a model based on the YOLOv8 algorithm that can automatically locate malignant regions in lung cancer cytological images. Evaluation results showed that 85.9% of images were correctly classified, achieving consistent and comparable performance with the results of experienced pathologists, which suggests that the model has the potential to make clinical-level decisions during initial diagnosis.

## Supporting information

S1 Fig Examples of model performance in SIC image diagnosis. (A) and (B) show correctly diagnosed malignant cells. (C) and (D) show correctly diagnosed benign cells. (E) and (F) show the misdiagnosis of malignant cells on bronchial epithelial and inflammatory cells. (G) and (H) show ignored malignant cells covered by inflammatory cells.
(PDF)

S1 Table Final histopathologic diagnoses of lung lesion samples. (PDF)

S2 Table Characteristics of previous artificial intelligence models for analyzing lung cancer cytological images. (PDF)

## References

[1] Sung H, Ferlay J, Siegel RL, Laversanne M, Soerjomataram I, Jemal A, et al. Global cancer statistics 2020: GLOBOCAN estimates of incidence and mortality worldwide for 36 cancers in 185 countries. CA Cancer J Clin. 2021;71: 209–249. doi: 10.3322/caac.21660 33538338

[2] Oudkerk M, Devaraj A, Vliegenthart R, Henzler T, Prosch H, Heussel CP, et al. European position statement on lung cancer screening. Lancet Oncol. 2017;18: e754–e766. doi: 10.1016/S1470-2045(17)30861-6 29208441

[3] Schabath MB, Cote ML. Cancer progress and priorities: Lung cancer. Cancer Epidemiol Biomarkers Prev. 2019;28: 1563–1579. doi: 10.1158/1055-9965.EPI-19-0221 31575553 PMC6777859

[4] Bhandari S, Pham D, Pinkston C, Oechsli M, Kloecker G. Timing of treatment in small-cell lung cancer. Med Oncol. 2019;36: 47. doi: 10.1007/s12032-019-1271-3 31025131

[5] Chen R, Manochakian R, James L, Azzouqa AG, Shi H, Zhang Y, et al. Emerging therapeutic agents for advanced non-small cell lung cancer. J Hematol Oncol. 2020;13: 58. doi: 10.1186/s13045-020-00881-7 32448366 PMC7245927

[6] Rivera MP, Mehta AC, Wahidi MM. Establishing the diagnosis of lung cancer: Diagnosis and management of lung cancer, 3rd ed: American College of Chest Physicians evidence-based clinical practice guidelines. Chest: American College of Chest Physicians evidence-based clinical practice guidelines. 3rd ed. 2013;143;Supplement: e142S–e165S. doi: 10.1378/chest.12-2353 23649436

[7] Jois DS, Mutreja D, Handa A, Moorchung N. Correlation between transbronchial lung biopsy and lung cytology. Rev Esp Patol. 2020;53: 75–78. doi: 10.1016/j.patol.2019.05.002 32199597

[8] McPherson D, Buchalter SE. The role of bronchoalveolar lavage in patients considered for open lung biopsy. Clin Chest Med. 1992;13: 23–31. doi: 10.1016/S0272-5231(21)00834-0 1582146

[9] Gayen S. Malignant pleural effusion: Presentation, Diagnosis, and Management. Am J Med. 2022;135: 1188–1192. doi: 10.1016/j.amjmed.2022.04.017 35576996

[10] Sakr L, Roll P, Payan MJ, Liprandi A, Dutau H, Astoul P, et al. Cytology-based treatment decision in primary lung cancer: Is it accurate enough? Lung Cancer. 2012;75: 293–299. doi: 10.1016/j.lungcan.2011.09.001 21975144

[11] Teramoto A, Tsukamoto T, Kiriyama Y, Fujita H. Automated classification of lung cancer types from cytological images using deep convolutional neural networks. BioMed Res Int. 2017;2017: 4067832. doi: 10.1155/2017/4067832 28884120 PMC5572620

[12] Dey P. The emerging role of deep learning in cytology. Cytopathology. 2021;32: 154–160. doi: 10.1111/cyt.12942 33222315

[13] Lecun Y, Bengio Y, Hinton G. Deep learning. Nature. 2015;521: 436–444. doi: 10.1038/nature14539 26017442

[14] Piantadosi G, Sansone M, Fusco R, Sansone C. Multi-planar 3D breast segmentation in MRI via deep convolutional neural networks. Artif Intell Med. 2020;103: 101781. doi: 10.1016/j.artmed.2019.101781 32143788

[15] Lin Q, Luo M, Gao R, Li T, Man Z, Cao Y, et al. Deep learning based automatic segmentation of metastasis hotspots in thorax bone SPECT images. PLOS ONE. 2020;15: e0243253. doi: 10.1371/journal.pone.0243253 33270746 PMC7714246

[16] Tan LK, McLaughlin RA, Lim E, Abdul Aziz YF, Liew YM. Fully automated segmentation of the left ventricle in cine cardiac MRI using neural network regression. J Magn Reson Imaging. 2018;48: 140–152. doi: 10.1002/jmri.25932 29316024

[17] Ferl GZ, Barck KH, Patil J, Jemaa S, Malamut EJ, Lima A, et al. Automated segmentation of lungs and lung tumors in mouse micro-CT scans. Iscience. 2022;25: 105712. doi: 10.1016/j.isci.2022.105712 36582483 PMC9792881

[18] Wang S, Yang DM, Rong R, Zhan X, Xiao G. Pathology image analysis using segmentation deep learning algorithms. Am J Pathol. 2019;189: 1686–1698. doi: 10.1016/j.ajpath.2019.05.007 31199919 PMC6723214

[19] Meng M, Zhang M, Shen D, He G, Guo Y. Detection and classification of breast lesions with You Only Look Once version 5. Future Oncol. 2022;18: 4361–4370. doi: 10.2217/fon-2022-0593 36519579

[20] J. R, S. D, R. G, A. F, ^editors. You Only Look Once: Unified, Real-Time Object Detection. 2016 IEEE Conference on Computer Vision and Pattern Recognition (CVPR); 2016:779–88.

[21] Jiang H, Hu F, Fu X, Chen C, Wang C, Tian L, et al. YOLOv8-Peas: A lightweight drought tolerance method for peas based on seed germination vigor. Front Plant Sci. 2023;14: 1257947. doi: 10.3389/fpls.2023.1257947 37841608 PMC10568755

[22] Rahman S, Rony JH, Uddin J, Samad MA. Real-time obstacle detection with YOLOv8 in a WSN using UAV aerial photography. J Imaging. 2023;9. doi: 10.3390/jimaging9100216 37888323 PMC10607597

[23] Rašić M, Tropčić M, Karlović P, Gabrić D, Subašić M, Knežević P. Detection and segmentation of radiolucent lesions in the lower jaw on panoramic radiographs using deep neural networks. Medicina (Kaunas). 2023;59. doi: 10.3390/medicina59122138 38138241 PMC10744511

[24] Xie X, Fu CC, Lv L, Ye Q, Yu Y, Fang Q, et al. Deep convolutional neural network-based classification of cancer cells on cytological pleural effusion images. Mod Pathol. 2022;35: 609–614. doi: 10.1038/s41379-021-00987-4 35013527 PMC9042694

[25] Redmon J, Farhadi A. YOLOv3: An incremental improvement. Report; 2018.

[26] Kingma DP, Adam BJ. A method for stochastic optimization [conference submission]; 2014.

[27] Xu X, Geng Q, Gao F, Xiong D, Qiao H, Ma X. Segmentation and counting of wheat spike grains based on deep learning and textural feature. Plant Methods. 2023;19: 77. doi: 10.1186/s13007-023-01062-6 37528413 PMC10394929

[28] Kendall M, Gibbons JD. Rank Correlation methods. 5th ed. New York: Oxford University Press; 1990.

[29] Jie H, Li S, Gang S; Proceedings of the IEEE Conference on Computer Vision and Pattern Recognition (CVPR), 2018: 7132–7141.

[30] Thakur N, Alam MR, Abdul-Ghafar J, Chong Y. Recent Application of Artificial Intelligence in Non-Gynecological Cancer Cytopathology: A Systematic Review. Cancers. 2022;14(14). doi: 10.3390/cancers14143529 35884593 PMC9316753

[31] Landau MS, Pantanowitz L. Artificial intelligence in cytopathology: A review of the literature and overview of commercial landscape. J Am Soc Cytopathol. 2019;8: 230–241. doi: 10.1016/j.jasc.2019.03.003 31272605

[32] Stenman S, Linder N, Lundin M, Haglund C, Arola J, Lundin J. A deep learning-based algorithm for tall cell detection in papillary thyroid carcinoma. PLOS ONE. 2022;17: e0272696. doi: 10.1371/journal.pone.0272696 35944056 PMC9362950

[33] Holmström O, Linder N, Kaingu H, Mbuuko N, Mbete J, Kinyua F, et al. Point-of-care digital cytology with artificial intelligence for cervical cancer screening in a resource-limited setting. JAMA Netw Open. 2021;4: e211740. doi: 10.1001/jamanetworkopen.2021.1740 33729503 PMC7970338

[34] Tsuneki M, Abe M, Kanavati F. Deep learning-based screening of urothelial carcinoma in whole slide images of liquid-based cytology urine specimens. Cancers. 2022;15. doi: 10.3390/cancers15010226 36612222 PMC9818219

[35] Teramoto A, Yamada A, Kiriyama Y, Tsukamoto T, Yan K, Zhang L, et al Automated classification of benign and malignant cells from lung cytological images using deep convolutional neural network. Inform Med Unlocked. 2019;16: 100205. doi: 10.1016/j.imu.2019.100205

[36] Teramoto A, Tsukamoto T, Yamada A, Kiriyama Y, Imaizumi K, Saito K, et al. Deep learning approach to classification of lung cytological images: Two-step training using actual and synthesized images by progressive growing of generative adversarial networks. Plos One. 2020;15: e229951. doi: 10.1371/journal.pone.0229951 32134949 PMC7058306

[37] Gonzalez D, Dietz RL, Pantanowitz L. Feasibility of a deep learning algorithm to distinguish large cell neuroendocrine from small cell lung carcinoma in cytology specimens. Cytopathology. 2020;31: 426–31. doi: 10.1111/cyt.12829 32246504

[38] Kim T, Chang H, Kim B, Yang J, Koo D, Lee J, et al. Deep learning-based diagnosis of lung cancer using a nationwide respiratory cytology image set: improving accuracy and inter-observer variability. Am J Cancer Res. 2023;13: 5493–503. 38058836 PMC10695775

[39] Zhao M, Wang W, Ren Q, Ni H, Xiao X, Ma L. Modified you-only-look-once model for joint source detection and azimuth estimation in a multi-interfering underwater acoustic environment. J Acoust Soc Am. 2023;153: 2393. doi: 10.1121/10.0017828 37092946

[40] Tulbure AA, Tulbure AA, Dulf EH. A review on modern defect detection models using DCNNs—Deep convolutional neural networks. J Adv Res. 2022;35: 33–48. doi: 10.1016/j.jare.2021.03.015 35024194 PMC8721352

[41] Zhang B, Li J, Bai Y, Jiang Q, Yan B, Wang Z. An improved microaneurysm detection model based on SwinIR and YOLOv8. Bioengineering (Basel). 2023;10. doi: 10.3390/bioengineering10121405 38135996 PMC10740408

[42] Sahoo PK, Gupta P, Lai YC, Chiang SF, You JF, Onthoni DD, et al. Localization of colorectal cancer lesions in contrast-computed tomography images via a deep learning approach. Bioengineering (Basel). 2023;10. doi: 10.3390/bioengineering10080972 37627857 PMC10451186

[43] Akoglu H. User’s guide to correlation coefficients. Turk J Emerg Med. 2018;18: 91–93. doi: 10.1016/j.tjem.2018.08.001 30191186 PMC6107969

[44] Tsukamoto T, Teramoto A, Yamada A, Kiriyama Y, Sakurai E, Michiba A, et al. Comparison of fine-tuned deep convolutional neural networks for the automated classification of lung cancer cytology images with integration of additional classifiers. Asian Pac J Cancer Prev. 2022;23: 1315–1324. doi: 10.31557/APJCP.2022.23.4.1315 35485691 PMC9375620

[45] Malapelle U, de Rosa N, Rocco D, Bellevicine C, Crispino C, Illiano A, et al. EGFR and KRAS mutations detection on lung cancer liquid-based cytology: A pilot study. J Clin Pathol. 2012;65: 87–91. doi: 10.1136/jclinpath-2011-200296 21945923

[46] McAlpine ED, Pantanowitz L, Michelow PM. Challenges developing deep learning algorithms in cytology. Acta Cytol. 2021;65: 301–309. doi: 10.1159/000510991 33137806

[47] Van Es SL, Greaves J, Gay S, Ross J, Holzhauser D, Badrick T. Constant quest for quality: Digital cytopathology. J Pathol Inform. 2018;9: 13. doi: 10.4103/jpi.jpi_6_18 29721361 PMC5907455

[48] Donnelly AD, Mukherjee MS, Lyden ER, Bridge JA, Lele SM, Wright N, et al. Optimal z-axis scanning parameters for gynecologic cytology specimens. J Pathol Inform. 2013;4: 38. doi: 10.4103/2153-3539.124015 24524004 PMC3908726

