# Supplementary material for: Automated recognition and segmentation of lung cancer cytological images based on deep learning
Source: PLoS One. 2025 Jan 31;20(1):e0317996. doi: 10.1371/journal.pone.0317996 (PMC11785301; doi:10.1371/journal.pone.0317996)
Supplement: S1 Fig — (A) and (B) show correctly diagnosed malignant cells. (C) and (D) show correctly diagnosed benign cells. (E) and (F) show the misdiagnosis of malignant cells on bronchial epithelial and inflammatory cells. (G) and (H) show ignored malignant cells covered by inflammatory cells. (PDF) [file pone.0317996.s001.pdf]

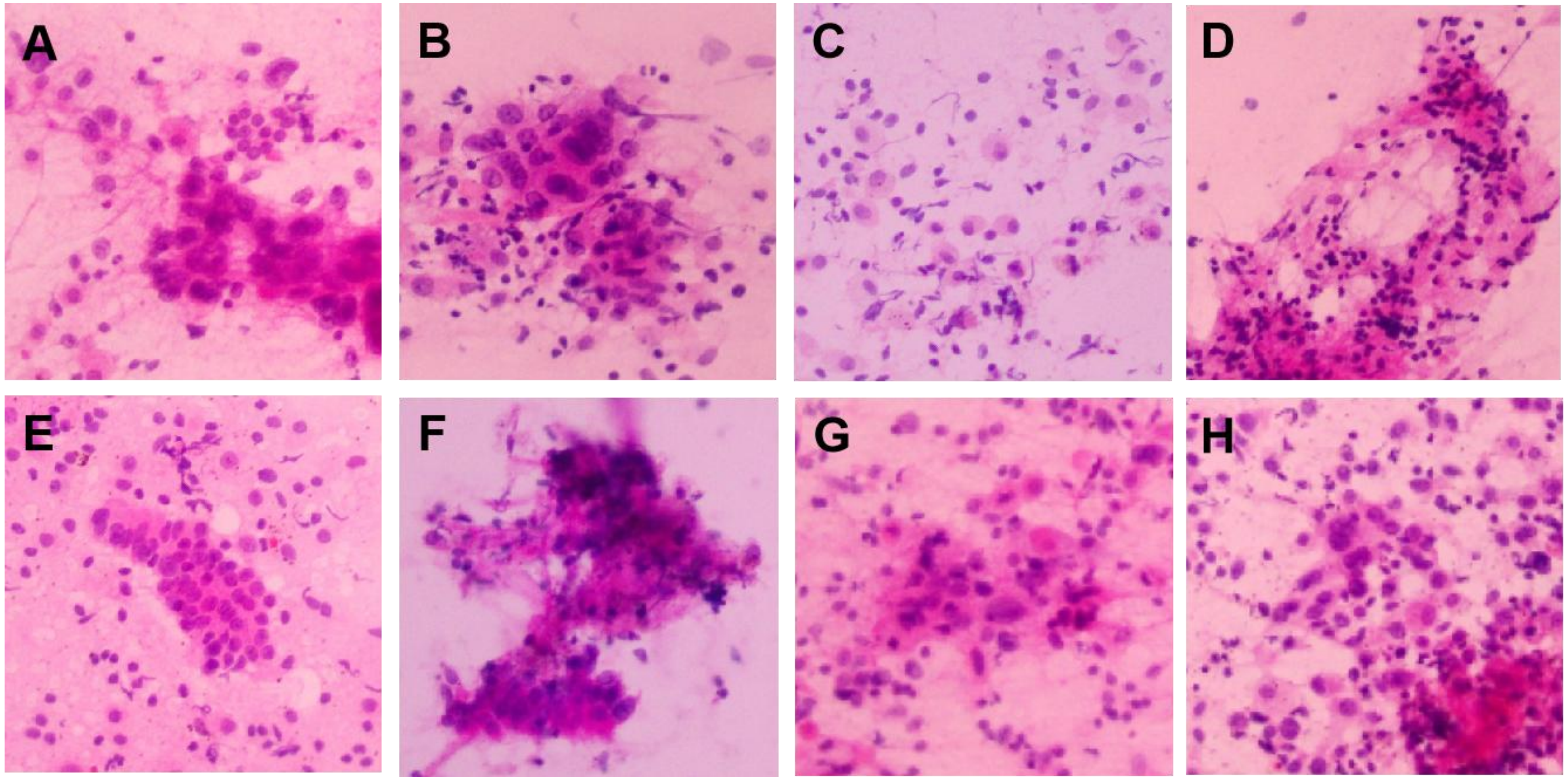

S1 Fig. Examples of model performance in SIC image diagnosis. (A) and (B) show correctly diagnosed malignant cells. (C) and (D) show correctly diagnosed benign cells. (E) and (F) show the misdiagnosis of malignant cells on bronchial epithelial and inflammatory cells. (G) and (H) show ignored malignant cells covered by inflammatory cells.
