# Supplementary material for: Automated recognition and segmentation of lung cancer cytological images based on deep learning
Source: PLoS One. 2025 Jan 31;20(1):e0317996. doi: 10.1371/journal.pone.0317996 (PMC11785301; doi:10.1371/journal.pone.0317996)
Supplement: S1 Table — (PDF) [file pone.0317996.s002.pdf]

S1 Table. Baseline characteristics of lung lesion samples.

	Total (n=405)
Age, Years	60.6±11. 9
Gender	
Male	167 (41.2)
Female	238 (58.8)
Size，cm	2.0±1.2
Histological type	
Malignant	362 (89.4)
Adenocarcinoma	320 (79.0)
Squamous cell carcinoma	27 (6.7)
Small cell carcinoma	1（0.2）
Large cell neuroendocrine carcinoma	3（0.7）
Sarcomatoid carcinoma	5（1.2）
Metastatic carcinoma	4（1.0）
Adenoid cystic carcinoma	1（0.2）
Mucoepidermoid carcinoma	1（0.2）
Benign	43（10.6）
Hamartoma	7 (1.7）
Pulmonary sclemsing pneumocytoma	2 (0.5）
Granulomatous inflammation/lesion	16 (4.0）
Fungal pneumonia	4 (1.0）
Non-specific inflammatory lesion	14 (3.5）
