# Supplementary material for: Automated recognition and segmentation of lung cancer cytological images based on deep learning
Source: PLoS One. 2025 Jan 31;20(1):e0317996. doi: 10.1371/journal.pone.0317996 (PMC11785301; doi:10.1371/journal.pone.0317996)
Supplement: S2 Table — (PDF) [file pone.0317996.s003.pdf]

S2 Table. Characteristics of previous artificial intelligence models using lung cancer cytological image analysis.

Author	Year	Task	Dataset	Model	Accuracy	Sensitivity	Specificity	AUC	[illegible] on with pathologi[illegible]
Teramoto	2017	[illegible] classification (ADC/SC[illegible]	76 cases (298 patches)	CNN	71.1%	NA	NA	NA	No
Teramoto	2019	classification (Benign/ Malignant[illegible]	46 cases (621 patches)	VGG-16	79.2%	89.3%	83.3%	93.2%	No
Teramoto	2020	classification (Benign/ Malignant[illegible]	60 cases (793 patches)	GAN	85.3%	85.4%	85.3%	90.1%	No
Gonzalez	2020	Subtypes classification (SCLC/LCNEC)	40 cases (114 WSIs)	Inception V3	NA	[illegible] (H&E) 100% (PAP) 100% (Diff-Q[illegible]	[illegible] (H&E) 85.7% (PAP) 87.5 (Diff-Q[illegible]	[illegible] (H&E) 100% (PAP) 100% (Diff-Q[illegible]	No
Xie	2022	classification (Benign/ Malignant[illegible]	404 cases (404 WSIs)	ResNet18	91.7%	87.5%	94.4%	95.3%	Yes
Kim	2023	Binary classification (Benign/ Malignant[illegible]	1273 WSIs	DenseNet 211	98.6% (H&E) 95.0% (PAP)	88.9% (H&E) 95.6% (PAP)	99.3% (H&E) 94.4% (PAP)	NA	Yes

Notes: ADC, adenocarcinoma; SCC, squamous cell carcinoma; SCLC, small cell lung cancer; LCNEC, large cell neuroendocrine carcinoma; WSI, whole slide image; CNN, convolutional neural network; GAN, generative adversarial network; NA, not available; H&E, hematoxylin and eosin; PAP, Papanicolaou; AUC, area under the receiver operating characteristic curve.
